# l-Palmitoylcarnitine potentiates plasmin and tPA to inhibit thrombosis

**DOI:** 10.1007/s13659-023-00413-z

**Published:** 2023-11-08

**Authors:** Juan Yang, Lina Cha, Yepeng Wang, Quan Zhang, Xiaopeng Tang, Jianlin Shao, Zilei Duan

**Affiliations:** 1 Department of Anesthesiology, First Affiliated Hospital of Kunming Medical University, Kunming, 650032 China; 2 State Key Laboratory of Drug Research, Shanghai Institute of Materia Medica, Chinese Academy of Sciences, Shanghai, 201203 China; 3 College of Life Sciences, Nanjing Agricultural University, Nanjing, 210095 China; 4 Small Molecule Drugs Sichuan Key Laboratory, Institute of Materia Medica, School of Pharmacy, Chengdu Medical College, Chengdu, 610500 China; 5 School of Basic Medicine, Qingdao University, Qingdao, 266071 Shandong China

**Keywords:** l-palmitoylcarnitine, Coagulation, Plasmin, Tissue-type plasminogen activator, Thrombosis

## Abstract

l-Palmitoylcarnitine (L-PC) is an important endogenous fatty acid metabolite. Its classical biological functions are involved in the regulations of membrane molecular dynamics and the β-oxidation of fatty acids. Decreased plasma long-chain acylcarnitines showed the association of venous thrombosis, implying anticoagulant activity of the metabolites and inspiring us to investigate if and how L-PC, a long-chain acylcarnitine, takes part in coagulation. Here we show that L-PC exerted anti-coagulant effects by potentiating the enzymatic activities of plasmin and tissue plasminogen activator (tPA). L-PC directly interacts with plasmin and tPA with an equilibrium dissociation constant (KD) of 6.47 × 10^–9^ and 4.46 × 10^–9^ M, respectively, showing high affinities. In mouse model, L-PC administration significantly inhibited FeCl_3_-induced arterial thrombosis. It also mitigated intracerebral thrombosis and inflammation in a transient middle cerebral artery occlusion (tMCAO) mouse model. L-PC induced little bleeding complications. The results show that L-PC has anti-thrombotic function by potentiating plasmin and tPA.

**Graphical Abstract:**

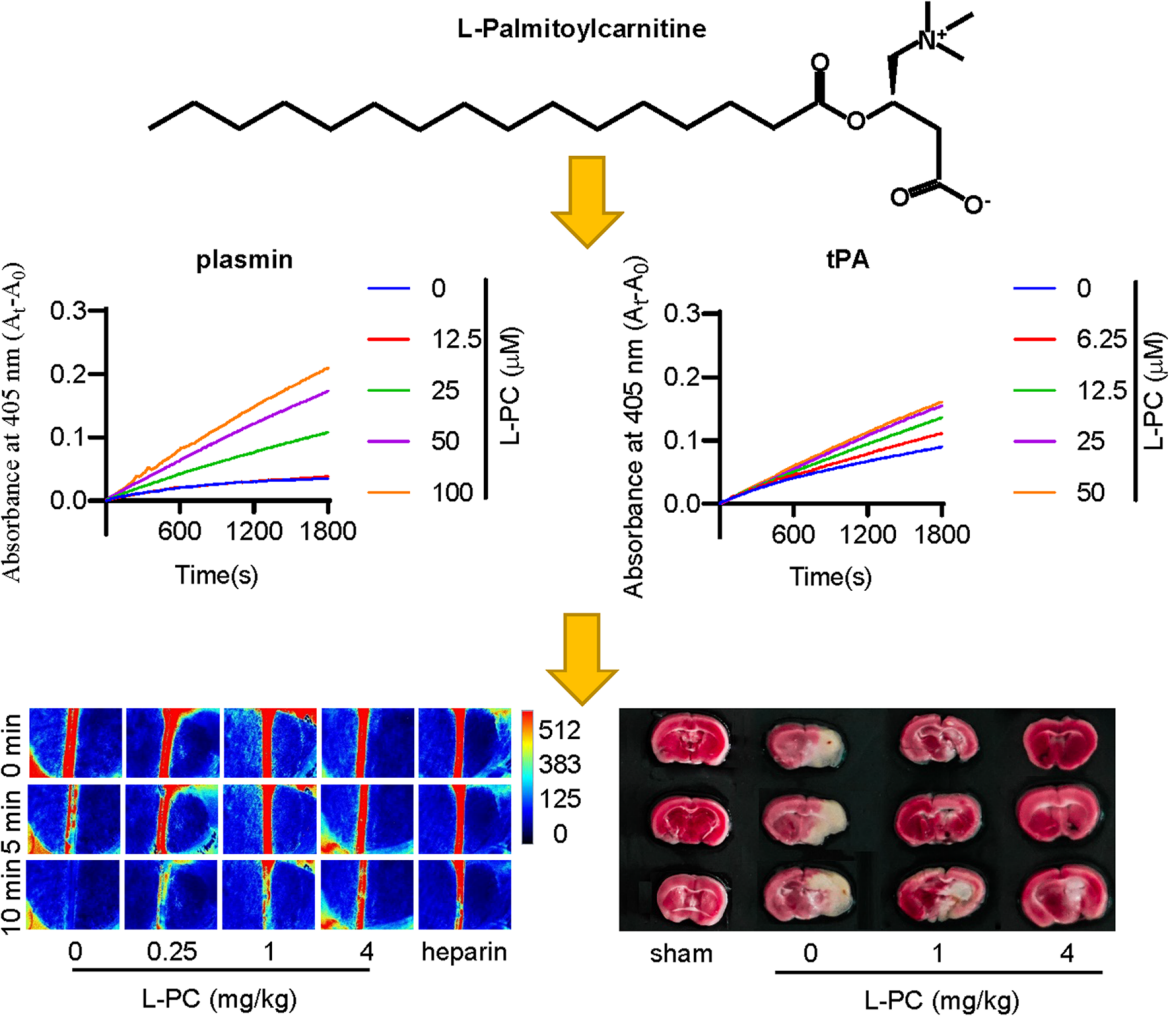

**Supplementary Information:**

The online version contains supplementary material available at 10.1007/s13659-023-00413-z.

## Introduction

L-PC is a well-known intermediate in mitochondrial fatty acid oxidation. L-PC is synthesized through the catalyzation of palmitoyl-CoA and l-carnitine (L-C), which is present in various food items and an essential nutrient for energy production and fatty acid metabolism [1]. L-C supplementation has also been shown to reduce serum C-reactive protein, a marker of systemic inflammation, and plasma fibrinogen in hemodialysis patients [2], although the underlying mechanism remains unclear. In comparison to L-C, the role of L-PC has been less extensively studied. L-PC has been implicated in the pathology of cardiac ischemia [3]. L-PC was found to be accumulated in the ischemic heart due to the inhibition of mitochondrial β-oxidation of fatty acid. It also induces membrane dysfunction in ischemic myocardium via Ca^2+^-dependent and -independent mechanisms [4, 5]. Moreover, it causes disorders of vascular endothelium [6, 7]. L-PC potentiates the functions of caspases to take part in apoptosis [8]. Therefore, the amphipathic metabolite L-PC, acts as one of key molecules in ischemia induced cell damage and induces elevation of intracellular Ca^2+^ level in various types of cells [9]. Some of remarkable physicochemical properties of L-PC, such as amphiphilicity and long-chain (> 10 carbons), may be involved in the pathogenesis of myocardial ischemia, which are unrelated to mitochondrial fatty acid metabolism [3]. The physicochemical properties of L-PC may contribute to lipid-clotting factor interactions to regulate the functions of clotting factors and blood coagulation [10]. However, it is unknown if and how L-PC affects coagulation.

In this study, we found the long-chain acylcarnitine L-PC has direct interactions with two enzymes plasmin and tPA, which are related to fibrin (ogen)olysis. L-PC potentiated the enzymatic activities of plasmin and tPA to promote fibrin (ogen)olysis. Mouse models showed anti-coagulant and anti-thrombotic functions of L-PC. The study revealed the regulatory function of L-PC on coagulation and intensified the paradigm for lipid-clotting factor interactions.

## Results

### L-PC promotes fibrin (ogen)olysis by potentiating plasmin and tPA

To determine whether L-PC has effect on coagulation, we initially evaluated the effects of L-PC on recalcification time (RT) of platelet-poor human plasma. As illustrated in Additional file 1: Figure S1A, stimulation with L-PC showed no effect on recalcification time. The coagulation cascade consists of extrinsic and intrinsic pathways, which can be assessed by prothrombin time (PT) and activated partial thromboplastin time (APTT) analysis, respectively. To confirm the effect of L-PC on coagulation, we further determined the influence of L-PC on PT and APTT. Similarly with the recalcification time, the results showed that L-PC stimulation did not significantly affect APTT (Additional file 1: Figure S1B) or PT (Additional file 1: Figure S1C).

**Fig. 1:**
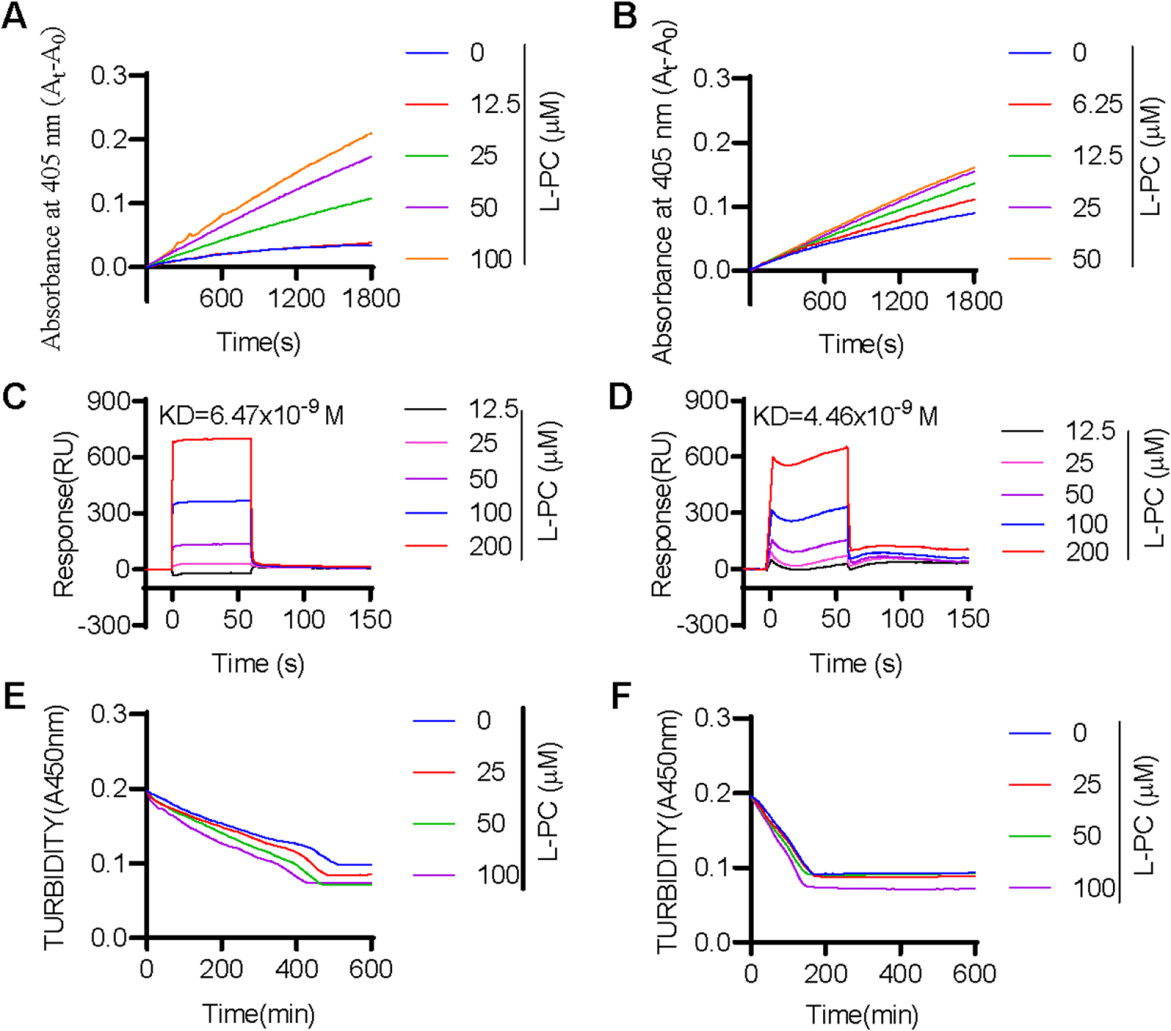
l-palmitoylcarnitine promotes fibrin(ogen)olysis by potentiating plasmin and tPA. Compared with the control group, L-PC enhanced the abilities of plasmin (**A**) and tPA (**B**) to hydrolyze their chromogenic substrates. Surface plasmon resonance (SPR) analysis of interaction between L-PC and plasmin (**C**) and tPA (**D**) on sensor CM5 chip. L-PC could bind to plasmin and tPA with equilibrium dissociation constants (KD) of of 6.47 × 10^–9^ and 4.46 × 10^–9^ M, respectively. L-PC enhanced the abilities of plasmin (**E**) and tPA (**F**) to hydrolyze their physiological substrates. Data are mean ± SD of at least three independent experiments.*p < 0.05, **p < 0.01, ***p < 0.001

We next evaluated the impact of L-PC on fibrinolysis by measuring the enzymatic activity of plasmin and tPA against their respective chromogenic substrates. Results showed that L-PC enhanced the enzymatic activity of both plasmin (Fig. 1A) and tPA (Fig. 1B) against their chromogenic substrates, respectively. We further explored the direct interactions of L-PC with plasmin and tPA using SPR. As shown in Fig. 1C and 1D, L-PC directly interacted with plasmin and tPA, with KD values of 6.47 × 10^–9^ and 4.46 × 10^–9^ M, respectively, showing high affinities.

We further explored the impact of L-PC on plasmin-mediated fibrinolysis by applying plasmin with or without L-PC to the surface of preformed clots, with fibrinolysis then monitored using a Cytation 3 Cell Imaging Multi-Mode Reader (Biotek). As shown in Fig. 1E, L-PC enhanced fibrin digestion in a dose-dependent manner. Given that L-PC also promoted the enzymatic activity of tPA, we next examined the effect of L-PC on fibrinolysis in the presence of plasminogen and tPA. Similar to plasmin-mediated fibrinolysis, L-PC enhanced tPA-plasminogen-mediated fibrinolysis (Fig. 1F). Collectively, these results suggest that L-PC facilitates fibrinolysis through the potentiation of plasmin and tPA activity.

### L-PC prevents thrombosis in vivo

**Fig. 2:**
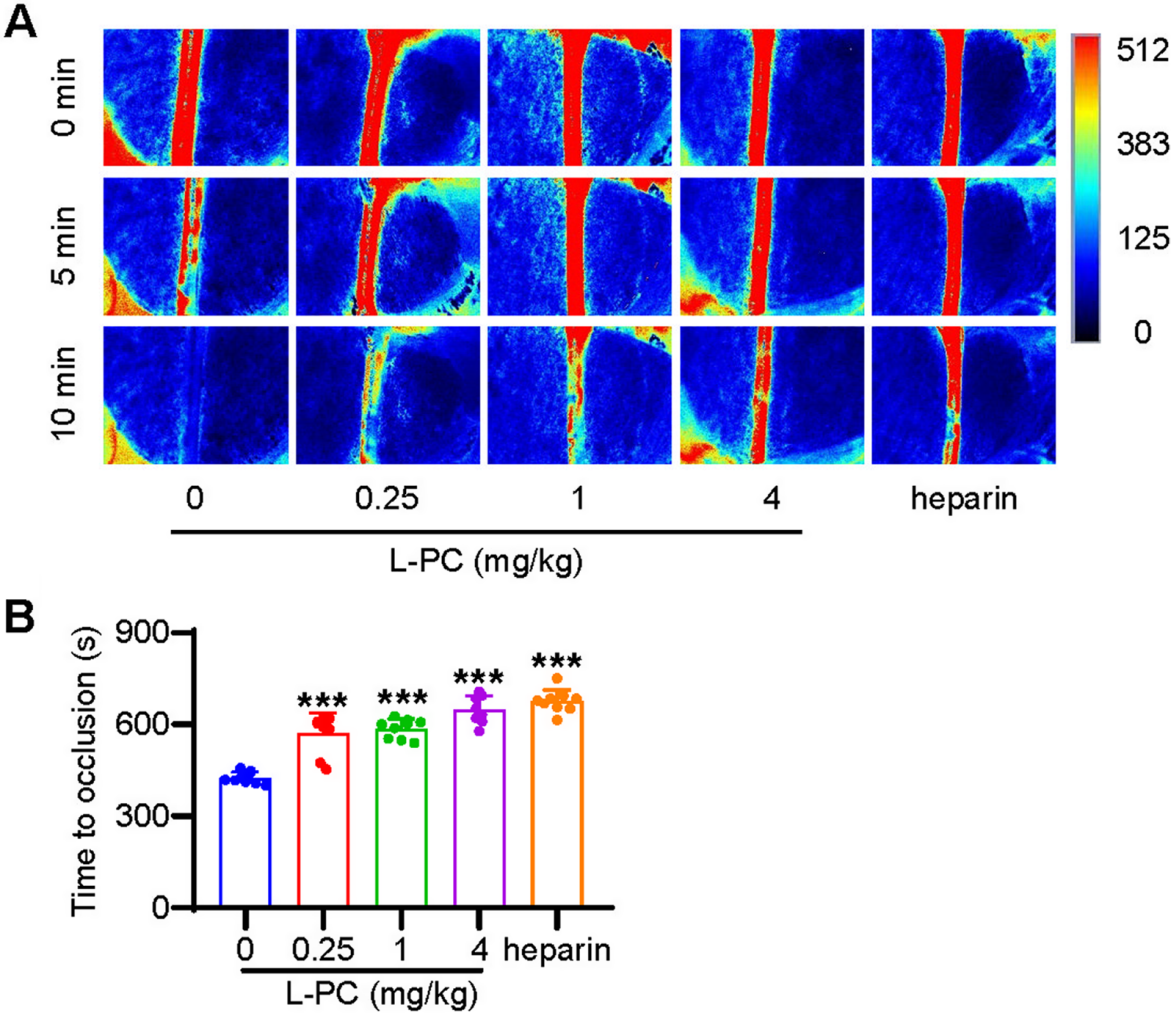
l-palmitoylcarnitine inhibits FeCl3-induced thrombosis in vivo. L-PC intravenous administration (0–4 mg/kg) inhibited thrombosis formation (**A**) and prolonged the time of arterial occlusion (**B**) in FeCl_3_-induced carotid artery thrombosis mouse model. Representative images of carotid artery blood flow at 0, 5, and 10 min (**A**), and statistical analysis of vascular occlusion time (**B**) of each group. Heparin was as the positive control. Data are mean ± SD of at least three independent experiments. ***p < 0.001

To assess the inhibitory effects of L-PC on thrombosis in vivo, an FeCl_3_-induced arterial thrombosis model was employed and carotid artery blood flow was examined using a laser perfusion imaging system. As shown in Fig. 2A, carotid artery blood flow was nearly undetectable at 5 min after injury and totally undetectable at 10 min after injury in the L-PC-untreated control group. However, in the L-PC-stimulated groups (0.25–4 mg/kg), the formation of FeCl_3_-induced arterial thrombosis was significantly inhibited. The inhibitory effect of L-PC (4 mg/kg) on thrombosis formation was comparable to that observed in the heparin-treated group. Additionally, L-PC treatment significantly prolonged the mean time for carotid artery occlusion (Fig. 2B). Notably, while the mean occlusion time for the L-PC-untreated group was 425.6 ± 18.21 s, this time was significantly extended by L-PC treatment (0.25, 1, 4 mg/kg) to 570.1 ± 62.73 s, 586.9 ± 29.80 s, and 647.8 ± 42.62 s, respectively. These findings suggest that L-PC exhibits a dose-dependent inhibition of thrombosis formation.

### L-PC protects mice from ischemic stroke

**Fig. 3:**
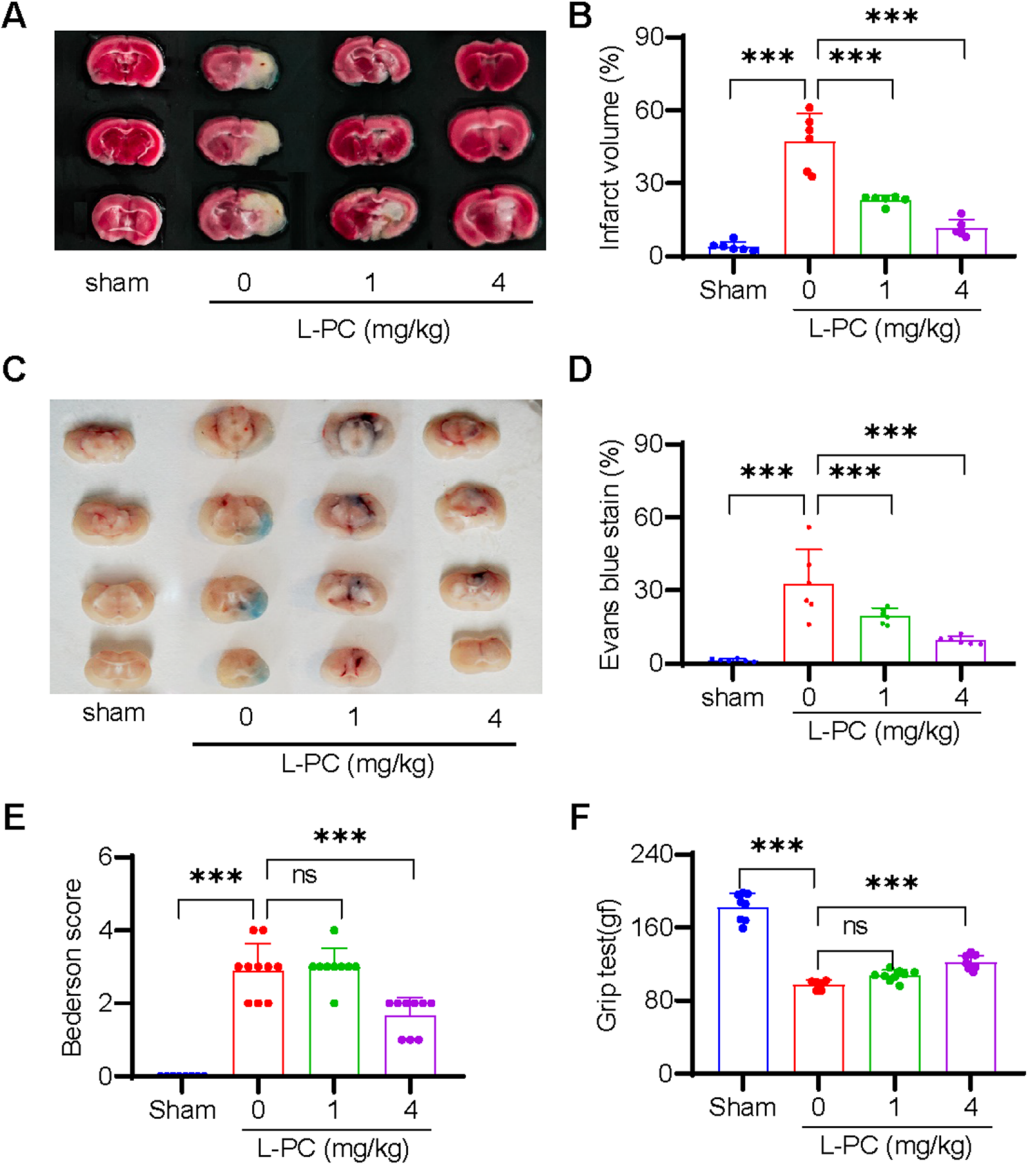
l-palmitoylcarnitine protects mice from ischemic stroke by counteracting intracerebral thrombosis in the tMCAO model. Representative sample of brain slices with 2% TTC staining **A** and Evans blue staining (**C**) after tMCAO, and the quantification of the ischemic lesion size **B** in **A** and the Evans blue staining area **D** in **C**. Bederson score **E** and grip test score **F** after tMCAO. Data are mean ± SD of at least three independent experiments. ***p < 0.001

Ischemic stroke (IS) is recognized as one of the leading causes of disability and death worldwide and is typically precipitated by the occlusion of cerebral vasculature following a thrombotic event [11]. Given the function of L-PC to facilitate fibrin(ogen)olysis and inhibit thrombosis as mentioned above, we explore anti-IS effects of L-PC by using a tMCAO mouse model to mimic IS. As illustrated in Fig. 3A, L-PC treatment (1 and 4 mg/kg) induced a significant and progressive dose-dependent decrease in infarct volumes. On average, the infarct volumes in the L-PC-treated groups (1 mg/kg: 23.25 ± 1.849%, 4 mg/kg: 11.47 ± 3.472%) were significantly smaller than that in the untreated group (47.38 ± 11.35%) (Fig. 3B). Moreover, to evaluate the dysfunction of the blood–brain barrier (BBB), we assessed Evans blue extravasation in the L-PC-treated groups (1 and 4 mg/kg) as well as the untreated group (0 mg/kg) following IS. As shown in Fig. 3C, L-PC administration prior to the induction of IS significantly attenuated the BBB dysfunction induced by cerebral ischemia–reperfusion. Consistently, the cerebral ischemia–reperfusion-induced area of Evans blue staining, indicative of BBB disruption, was significantly reduced under L-PC treatment (0 mg/kg: 32.69 ± 14.06%, 1 mg/kg: 19.66 ± 3.128%, 4 mg/kg: 9.59 ± 1.657%) (Fig. 3D). Furthermore, the smaller infarct volumes observed in the L-PC-treated groups were associated with improved functional outcomes (Fig. 3E: Bederson score; Fig. 3F: grip test score). Taken together, these findings suggest that L-PC provides marked protection against IS.

### L-PC inhibits IS-induced inflammation

**Fig. 4:**
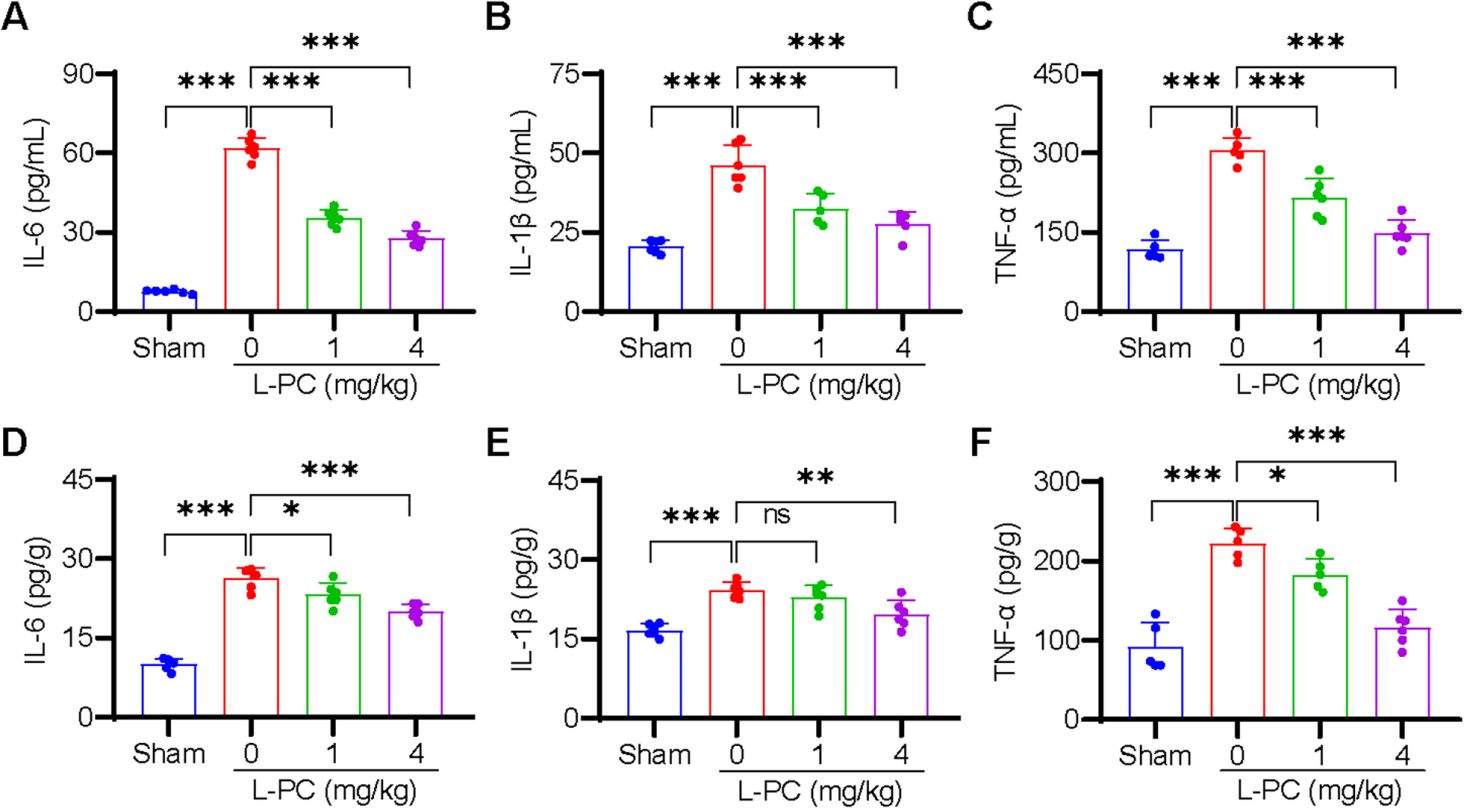
l-palmitoylcarnitine inhibits inflammation in the tMCAO mouse model. Levels of cytokines of serum (**A**-**C**) and brain (**D**-**F**) after tMCAO with or without intravenous treatment of L-PC (1, 4 mg/kg) were analyzed by ELISA. Higher levels of IL-6 (**A**, **D**), IL-1β (**B**, **E**), TNFα (**C**, **F**) in serum and brain induced by tMCAO were significantly attenuated with the treatment with L-PC. Data are mean ± SD of at least three independent experiments.*p < 0.05, **p < 0.01, ***p < 0.001

The inflammatory response is known to be potentiated by clots and commonly occurs after IS [12, 13]. Thus, we further investigated the effects of L-PC treatment on IS-induced inflammation. As illustrated in Fig. 4, compared to the sham-operated mice, the serum levels of IL-6, IL-1β, and TNF-α were significantly increased in the L-PC-untreated tMCAO group. Conversely, the levels of IL-6 (Fig. 4A), IL-1β (Fig. 4B), and TNF-α (Fig. 4C) were significantly decreased in the L-PC-treated tMCAO groups. We also evaluated inflammation levels in the brain, and analogous to the serum results, significant reductions in tMCAO-induced IL-6 (Fig. 4D), IL-1β (Fig. 4E), and TNF-α (Fig. 4F) levels were observed following L-PC treatment. Collectively, these findings suggest that L-PC effectively inhibits inflammation triggered by IS.

### L-PC induces little bleeding complications

Bleeding is considered the most serious side effect of clinical anti-thrombotic drugs. To assess the potential bleeding complications associated with L-PC, we conducted a mouse tail bleeding test. As illustrated in Figure S2, L-PC exhibited minimal bleeding complications at the higher concentrations of 1 and 4 mg/kg, which was significantly lower compared to the heparin-treated group. This suggests that L-PC has a favorable bleeding profile as a potential anti-thrombotic agent.

## Discussion

Thrombosis is implicated in the pathogenesis of many diseases, and strategies involving anticoagulation or fibrinolysis are crucial for the treatment and prevention of thrombotic disorders. In this study, we found that the endogenous metabolic molecule L-PC possesses potent anticoagulant properties by enhancing the enzymatic activity of plasmin and tPA, which was further confirmed by the significant inhibitory effects on the development of thrombosis and IS in vivo. The anticoagulant properties are mediated by the lipid-clotting factor interactions between L-PC and plasmin/tPA with high affinities.

Both L-PC and L-C play important roles in mitochondrial β-oxidation, which is essential for maintaining energy homeostasis in the human body [14]. As illustrated in Fig. 1, L-PC could directly bind to two enzymes (plasmin and tPA) that are responsible for thrombolysis and fibrin(ogen)olysis. Through high-affinity interactions with plasmin (KD: 6.47 nM) and tPA (KD: 4.46 nM), L-PC significantly enhanced their hydrolytic abilities. As illustrated in Figs. 2 and 3, in vivo mouse models further confirmed the anticoagulant functions of L-PC. Figure 2 shows that in the model of FeCl_3_-induced arterial thrombosis, even 0.25 mg/kg L-PC administration prolonged the occlusion time from 310 to 520 s, indicating significantly anti-thrombotic effects. In the tMCAO mouse model, 1 mg/kg L-PC decreased the infract volume by 50% in the brains and significantly inhibited the production of inflammatory factors in the serum and brain tissues, showing potential anti-IS ability.

Fatty acids demonstrate intricate interactions with the hematological system, with unsaturated fatty acids promoting fibrinolysis and saturated fatty acids displaying inhibitory effects [15]. There are hundreds of minor lipids in plasma, many of which likely modulate clotting factor activities and affect thrombosis risk. For example, some minor abundance plasma lipids are found to modulate thrombin generation via direct effects on factor Xa, and some acylcarnitine lipids contained anticoagulant functions [10]. Gamma carboxyglutamic acid (Gla) is a common domain in many vitamin K-dependent coagulation factors, such as prothrombin, factor X, factor IX, factor VII, protein C, and protein S. Gla domain is an interface for interacting with fatty acids [16–18]. The current study identified strong interactions between L-PC and plasmin and t-PA. Further studies are necessary to determine the interface responsible for the interactions. Although many minor plasma lipids have been identified, only a few studies have been done to investigate the role of individual fatty acids in hemostasis [15]. More investigations of procoagulant or anticoagulant effects of fatty acids and their derivatives will provide valuable insights into the paradigm for lipid clotting factor interactions.

## Conclusions

In conclusion, we identified L-PC as an endogenous potentiator of the fibrinolytic system by enhancing the functions of plasmin and t-PA. Its administration showed significant therapeutic effects on thrombotic disorders, suggesting a potential to achieve beneficial effects by L-PC supplementation through dietary adjustments.

## Experimental section

### Blood coagulation time assay

Blood recalcification time was measured in accordance with previous research [19]. Briefly, 19 μL of fresh plasma obtained from healthy human donors was mixed with 1 μL of L-PC (HY-113147A, MedChemExpress, USA. final concentrations of 0, 25, and 50 μM) and 40 μL of HEPES buffer (20 mM HEPES, 150 mM NaCl, pH 7.4) in 96-well plate. The plates were then incubated for 10 min in a 37 °C incubator, followed by the addition of 40 μL of preheated (37 °C) CaCl_2_ solution (25 mM). The kinetic program was recorded using a Cytation 3 Cell Imaging Multi-Mode Reader (Biotek) to monitor absorbance at 650 nm every 30 s for 30 min.

Activated partial thromboplastin time (APTT) is used to evaluate the functionality and integrity of the intrinsic and common coagulation pathways, while prothrombin time (PT) is used to detect the functionality and integrity of the extrinsic and common coagulation pathways. Here, we determined the effects of L-PC on APTT and PT using commercial kits according to the provided directions. Briefly, for the APTT assay, 50 μL of APTT reagent (Beijing Reagan Biotechnology, China) and 50 μL of plasma were mixed with 5 μL of L-PC at different concentrations (0, 25, 50 μM). Clotting time was then recorded after the addition of 50 μL of preheated (37 °C) CaCl_2_ (25 mM), with absorbance monitored at 650 nm using a Cytation 3 Cell Imaging Multi-Mode Reader (Biotek). For the PT assay, 50 μL of plasma was mixed with 5 μL of L-PC at different concentrations (0, 25, 50 μM). Clotting time was then recorded after the addition of 100 μL of PT reagent (Nanjing Jiancheng Bioengineering Institute, China) preheated at 37 °C for 15 min, with then absorbance monitored at 650 nm using a Cytation 3 Cell Imaging Multi-Mode Reader (Biotek).

### Enzyme kinetic assays

The enzymes used in this study were tissue-type fibrinogen activator and plasmin, with chromogenic substrates used for the enzyme kinetic experiments according to the method reported before [20]. In brief, the enzymatic reactions were conducted in 50 mM Tris–HCl buffer (pH 7.8) at 37 °C. Initially, the proteases (plasmin: HPlasmin, Enzyme Research Laboratory, USA (30 nM); tPA: T0831, Sigma, USA (20 nM)) and serial dilutions of L-PC (6.25–100 μM) were incubated in buffer at 37 °C for 5 min, followed by the addition of their corresponding chromogenic substrates (CS-41, Hyphen Biomed, France (0.2 mM) for plasmin; CS-05, Hyphen Biomed, France (0.2 mM) for tPA) to initiate the reaction. The rate of substrate hydrolysis was monitored continuously at 405 nm.

### Fibrin polymer lysis assay

The fibrin polymer lysis assay was used to examine the effects of L-PC on the activity of plasmin and tPA towards their physiological substrates according to the method reported before[21]. Fibrin clots were formed by incubating fibrinogen with thrombin in buffer (50 mM Tris–HCl, pH 7.4, 100 mM NaCl, and 5 mM CaCl_2_) at room temperature until turbidity reached a stable level. Plasmin or tPA plus plasminogen in the presence or absence of L-PC was gently layered on top of the fibrinogen polymer reaction mixture, with changes in turbidity at 450 nm monitored using a Cytation 3 Cell Imaging Multi-Mode Reader (Biotek).

### Surface plasmon resonance (SPR) analysis

SPR analysis was performed according to the manufacturer’s instructions. In brief, a CM5 sensor chip (29149603, Cytiva, USA) was first activated with 0.4 M 1-ethyl-3-(3-dimethylaminopropyl) carbodiimide hydrochloride and 10 mM N-hydroxysuccinimide at a flow rate of 5 μL/min for 20 min. Plasmin (HPlasmin, Enzyme Research Laboratory, USA) or tPA (HY-P71051, MedChemExpress, USA), diluted to a concentration of 100 μg/mL using sodium acetate buffer (10 mM, pH 5), flowed across the activated surface, allowing coupling with the chip until a target response value (RU) of 12 000 was reached. The remaining activated sites on the chip were blocked by 75 μL of ethanolamine (1 M, pH 8.5). Real-time detection was recorded using a Biacore S200 instrument (USA) at a flow rate of 10 μL/min. Subsequently, to detect the equilibrium dissociation constant (KD), serially diluted L-PC (12.5, 25, 50, 100, 200 μM in 0.01 M phosphate-buffered saline (PBS), pH 7.4) was applied to analyze its interactions with plasmin or tPA coupled on the chip surface using the BIA evaluation program.

### Bleeding time test

Male C57BL/6 mice (6 weeks old) were randomly divided into five groups (n = 6 per group): saline-treated group, three L-PC-treated groups (concentrations of 0.25, 1, 4 mg/kg), and sodium heparin-treated positive control group (2 500 U/kg). After 10-min intravenous administration of the respective drugs via the tail vein, the tails were cut at a distance of 2 mm from the tip and immersed in saline at 37 °C. The time taken for continuous blood flow to cease was recorded as bleeding time.

### FeCl_3_‑induced mouse thrombosis model

Male C57BL/6 J mice (6 weeks old) were anesthetized with 3% pentobarbital sodium (80 mg/kg) and fixed in a supine position. The left carotid artery (CCA) was exposed through a midline neck incision. Subsequently, four groups were established, each receiving different concentrations of L-PC (0, 0.25, 1, 4 mg/kg) administered via tail vein injections. After a 10-min interval, carotid arterial thrombosis was induced by placing a filter paper disc (diameter 5.0 mm) soaked in 10% FeCl_3_ onto the artery for 2 min. Blood flow was monitored using a laser-speckle blood flow imaging system (RFLSI Pro; RWD Life Science).

### Transient MCAO (tMCAO)

#### Assessment of functional outcome

Male C57BL/6 J mice (6 weeks old) were anesthetized with 3% pentobarbital sodium (80 mg/kg) and fixed in a supine position. The skin along the midline of the neck was incised to expose the external and internal carotid arteries. A standardized silicon rubber-coated nylon monofilament (6023910PK10; Doccol, Sharon, MA, USA) was carefully inserted into the left common carotid artery and advanced via the internal carotid artery to occlude the origin of the left middle cerebral artery, inducing ischemia. Subsequently, four groups were established, each receiving different concentrations of L-PC (0, 0.25, 1, or 4 mg/kg) administered 10 min prior to reperfusion. Reperfusion was initiated by removing the occluding filament 60 min after its insertion. After 24 h of modeling, neurological assessments, including Bederson score and grip test, were performed following previously established protocols [22].

#### Measurement of infarct size and volume

The mice were sacrificed 24 h after tMCAO and five 2-mm-thick coronal brain sections were obtained. The brain slices were stained with 2% 2,3,5-triphenyltetrazolium chloride (TTC, Sigma, USA) in a dark environment at 37 °C for 10 min. TTC reacts with intact mitochondrial respiratory enzymes, resulting in the unaffected brain regions appearing bright red and the infarct areas exhibiting a pale coloration. The extent of infarction in the brain sections was estimated by calculating the pale area. The infarct volume ratio was quantified using Image Pro Plus v6.0 (Media Cybernetics, Bethesda, MD, USA).

#### Evans blue staining

Mice were injected with 2% Evans blue (R31047, Yuanye, China) in the tail vein 2 h before sacrifice. Brain sections were taken to calculate the area of Evans blue staining using Image Pro Plus v6.0 (Media Cybernetics, Bethesda, MD, USA).

#### Enzyme-linked immunosorbent assay (ELISA)

Male C57BL/6 J mice (6 weeks old) were sacrificed 24 h after tMCAO, followed by the collection of brain tissue and blood. Mouse IL-1β ELISA kit (DG30045M-96 T, Dogesce, China), IL-6 ELISA kit (DG30062M-96 T, Dogesce, China), and TNF-α ELISA kit (DG30048M-96 T, Dogesce, China) were used to detect the levels of inflammatory factors in brain tissue and blood samples according to the manufacturer’s instructions.

### Statistical analyses

Data obtained from independent experiments are presented as mean ± standard deviation (SD). For normal continuous variables, one-way analysis of variance (ANOVA) was used. All analyses were conducted using GraphPad Prism v5. Asterisks represent p-value classifications: * *p* < 0.05; ** *p* < 0.01, and *** *p* < 0.001.

### Supplementary Information

**Additional file 1:** **Fig. S1. **Effects of L-PC on recalcification time, APTT and PT. Compared with the control group, the recalcification time (A) was similar with or without the stimulation by L-PC at the concentration of 25 and 50 μM. Using APTT and PT kit, effects of L-PC on coagulation cascade was determined. Compared with the L-PC untreated group, the APTT (B) and PT (C) were showed no significant difference after stimulated with L-PC with the concentration of 25 and 50 μM. **Fig. S2. **Effects of L-PC on bleeding. Mouse tail bleeding model was created, which was used to determine the bleeding risk of L-PC. At the higher concentration (1 and 4 mg/kg), L-PC showed minimal bleeding complications, which was much lower compared to that of the heparin-treated group. Data are mean ± SD of at least three independent experiments. **p < 0.01, ***p < 0.001.

## Data Availability

All the data supporting the findings of this study are available within the article and/or its supplementary materials.
