# Supplementary material for: l-Palmitoylcarnitine potentiates plasmin and tPA to inhibit thrombosis
Source: Nat Prod Bioprospect. 2023 Nov 8;13(1):48. doi: 10.1007/s13659-023-00413-z (PMC10632336; doi:10.1007/s13659-023-00413-z)
Supplement: Supplementary file 1 — Additional file 1: Fig. S1. Effects of L-PC on recalcification time, APTT and PT. Compared with the control group, the recalcification time (A) was similar with or without the stimulation by L-PC at the concentration of 25 and 50 μM. Using APTT and PT kit, effects of L-PC on coagulation cascade was determined. Compared with the L-PC untreated group, the APTT (B) and PT (C) were showed no significant difference after stimulated with L-PC with the concentration of 25 and 50 μM. Fig. S2. Effects of L-PC on bleeding. Mouse tail bleeding model was created, which was used to determine the bleeding risk of L-PC. At the higher concentration (1 and 4 mg/kg), L-PC showed minimal bleeding complications, which was much lower compared to that of the heparin-treated group. Data are mean ± SD of at least three independent experiments. **p < 0.01, ***p < 0.001. [file 13659_2023_413_MOESM1_ESM.docx]

**Supplementary materials**

**Supplementary Figures and Figure legends**

**
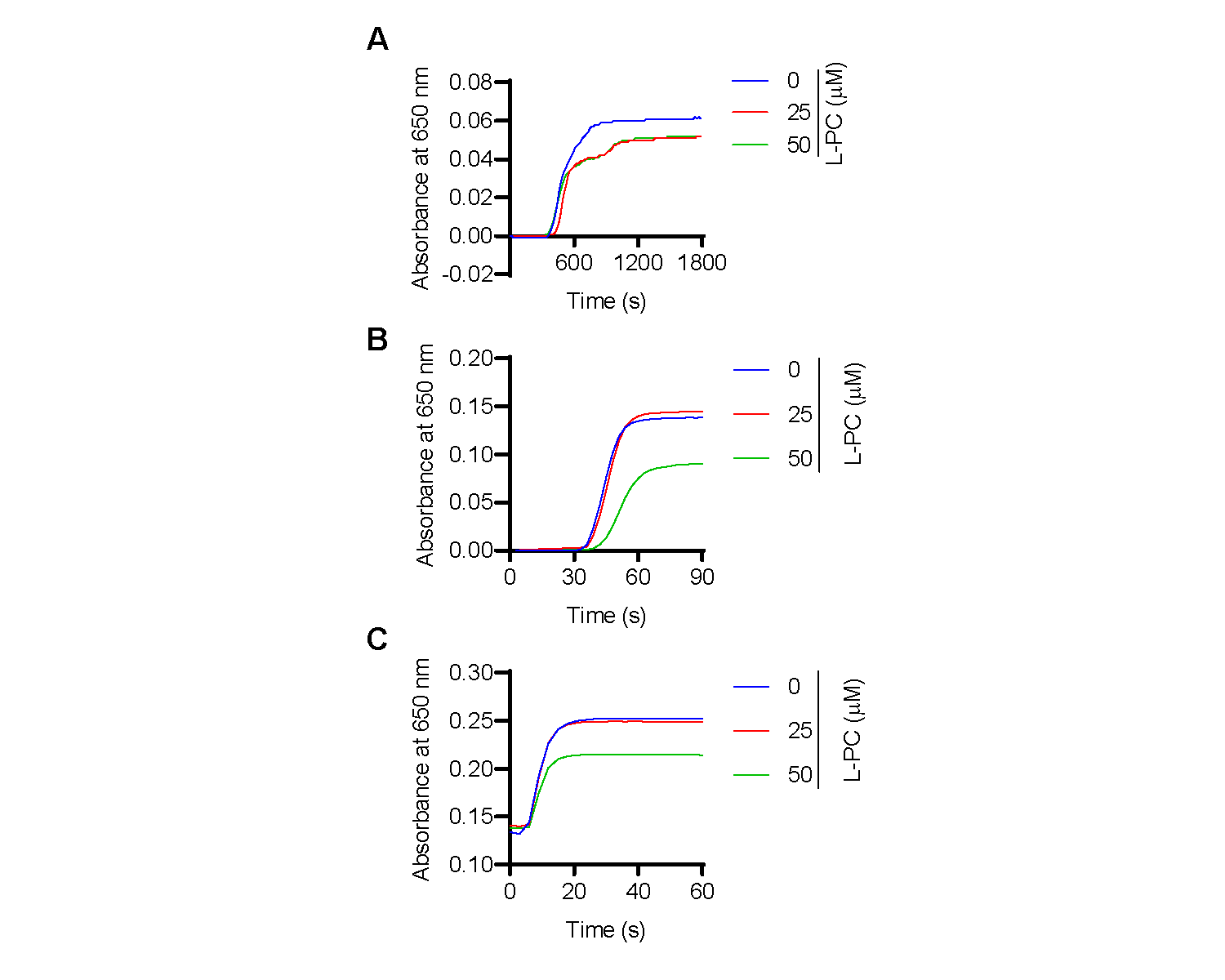
**

**Fig. S1. Effects of L-PC on recalcification time, APTT and PT.** Compared with the control group, the recalcification time (A) was similar with or without the stimulation by L-PC at the concentration of 25 and 50 μM. Using APTT and PT kit, effects of L-PC on coagulation cascade was determined. Compared with the L-PC untreated group, the APTT (B) and PT (C) were showed no significant difference after stimulated with L-PC with the concentration of 25 and 50 μM.

**
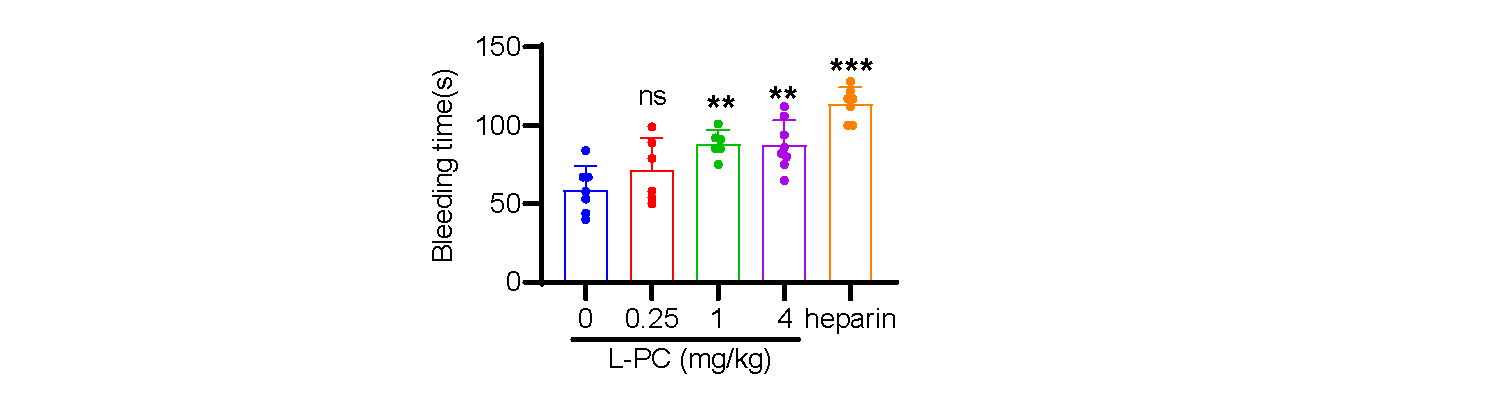
Fig. S2. Effects of L-PC on bleeding.** Mouse tail bleeding model was created, which was used to determine the bleeding risk of L-PC. At the higher concentration (1 and 4 mg/kg), L-PC showed minimal bleeding complications, which was much lower compared to that of the heparin-treated group. Data are mean ± SD of at least three independent experiments. ***p* < 0.01, ****p* < 0.001.
